# A Machine Learning Approach for Predicting Long-term Care Needs and Identifying Risk Factors Among Older Adults in Japan

**DOI:** 10.24546/0100498772

**Published:** 2025-12-12

**Authors:** HISATAKA ANEZAKI, MAMORU HIROE, MICHIYO KAWAI, AYAKO FUJIWARA, YUICHI NAKATA, YOSHIHARU MIYATA, HIROAKI MASUDA, AKIRA MATSUMOTO, SHINICHI OKATA, HISAKO IZUMI, KEN NAONO, YOICHI KUREBAYASHI

**Affiliations:** 1 Department of Artificial Intelligence and Digital Health Sciences, Kobe University Graduate School of Medicine, Kobe, Japan; 2 Graduate School of Science, Technology and Innovation, Kobe University, Kobe, Japan; 3 Research and Development Group, Hitachi, Ltd., Tokyo, Japan; 4 Government and Public Corporation Information Systems Division, Hitachi, Ltd., Kobe, Japan; 5 Department of Public Health, Kobe University Graduate School of Health Sciences, Kobe, Japan

**Keywords:** Machine learning, Long-term care needs, Risk prediction, XGBoost, Healthcare data

## Abstract

A machine learning model using Extreme Gradient Boosting (XGBoost) was developed to predict long-term nursing care needs among older adults, based on comprehensive claims and health checkup data from Japan’s public insurance system. The model demonstrated strong predictive performance (AUC: 0.878; sensitivity: 0.784; specificity: 0.820) on the test dataset, supporting its use for early identification of high-risk individuals. Key risk factors identified through permutation importance and marginal effect analyses included advanced age, prior care needs, neurological and gastrointestinal diseases, as well as specific medical procedures and medications. In contrast, factors such as joint replacement surgery and the use of preventive care services were associated with lower risk. Lifestyle and biochemical indicators, including slower gait speed and low LDL cholesterol, also significantly influenced risk. Constipation, osteoporosis, and lower back pain had relatively small marginal effects, but was associated with a high incidence rate. This model provides a valuable tool for extending healthy life expectancy and optimizing long-term care planning in aging populations, supporting both public health policy and personalized prevention.

## INTRODUCTION

As society continues to age rapidly, extending healthy life expectancy (HLE) has become a globally recognized public health priority. According to the 2020 United Nations report on aging (1), 703 million individuals were aged 65 years or older in 2019, comprising approximately 9% of the global population. This number is projected to double to 1.5 billion by 2050, representing 16% of the global population. The increase in life expectancy leads to a greater number of elderly individuals suffering from age-related diseases and disability, and imposes a large economic burden on individuals and societies (2–4). This demographic shift presents serious public health challenges, necessitating the development of effective strategies to promote health and maintain functional independence among older adults. These efforts strategies are essential not only for enhancing the quality of life among the aging population but also for addressing the substantial resources needed to support those who have lost their independence in daily activities (5, 6).

In this context, significant attention has been directed towards developing a tool capable of predicting the long-term nursing care needs among the elderly. Such a tool could greatly facilitate early interventions by healthcare professionals aimed at extending HLE for the elderly, thereby offering substantial social value. Machine learning (ML) is one of the most promising approaches for developing risk prediction models, leveraging the growing availability of diverse data in the healthcare field (7, 8). Recent studies, for instance, have demonstrated the advantages of machine learning in detecting, predicting, and classifying frailty in elderly individuals (9, 10). Nevertheless, the development of predictive machine learning models that can individually assess the risk of needing long-term nursing care remains highly challenging due to the complexity of its underlying causes. Beyond frailty, factors such as chronic diseases, lifestyle, and living conditions also impact HLE deterioration (11, 12). Therefore, comprehensive analysis of medical, long-term care, and health checkup data is essential for accurately assessing and addressing this multifaceted issue.

In the present study, we developed a machine learning model to predict individual risk of future nursing care needs among older adults, utilizing comprehensive claims and health checkup data from the Japanese public health insurance system. Furthermore, we conducted an extensive analysis to identify actionable risk factors, with the aim of supporting strategies to extend healthy life expectancy and mitigate the burden of long-term care at both individual and societal levels.

## MATERIALS AND METHODS

### Subjects of Analysis

Of approximately 380,000 residents aged 65 years or older who were eligible for Kobe City’s community-based integrated care system (CBICS) (13), 284,660 individuals were included in the analysis after excluding those with Long-Term Care Level 2 or higher certification during the baseline period (April 2016 to December 2017), as well as those who died or moved out of the city during the study period (April 2016 to December 2019). Under CBICS, care needs are classified into seven levels based on the type and extent of services necessary to support daily living, i.e., Support Required Levels 1 and 2, and Care Required Levels 1 through 5 (14). Certification of care levels is determined by physicians and long-term care professionals using assessments based on five domains: physical function and mobility, activities of daily living (ADL), cognitive function, mental and behavioral status, and social adaptation. Care Level 2 is defined as the threshold at which an individual is considered unable to independently perform most aspects of daily life and is recognized as the end of a healthy life (15).

The Kobe City Health Bureau provided this study with record-linked anonymized data used in this study, including the medical and caregiving claims, long-term care certification assessments, as well as health checkup records, sourced from the National Community-based Health Insurance System.

### Preparation of Training and Test Dataset

The original dataset contained 13,273 variables, including ICD-10-based diagnostic codes, medical procedures, prescribed medications, elderly care services, and health checkup data. All variables were binarized for use as explanatory features in machine learning analyses. Specifically, each variable was coded as 1 if at least one corresponding record was present during the 21-month baseline period, and as 0 if no such records were found. Continuous variables, such as age and laboratory test values, were categorized and then converted into binary variables. Variables with fewer than 10 occurrences were excluded to mitigate the risk of re-identification associated with rare conditions.

The presence or absence of certification for Care Level 2 or higher, as recorded in the long-term care certification assessment, was used as the dependent variable. For the purpose of machine learning, individuals certified at Care Level 2 or higher during the 2-year observation period following the baseline period were coded as 1, while those below this level were coded as 0.

The dependent variable was linked to a set of individual-level explanatory variables measured during the baseline period to construct two datasets: a training dataset for supervised machine learning and a test dataset for evaluating the predictive performance of the developed model. Specifically, the full cohort of 284,660 individuals was randomly divided into two subsets: a training pool (n = 213,098) and a test pool (n = 71,562). In both pools, 5.6% of individuals were certified as Care Level 2 or higher during the observation period. To mitigate bias due to class imbalance in machine learning, stratified under-sampling was applied to the training pool such that 49.9% of the resulting training dataset consisted of individuals certified as Care Level 2 or higher after the baseline period. As a result, the final training dataset comprised 23,878 individuals.

### Machine Learning

Given that the total number of variables related to diagnoses and medical procedures in medical claims exceeded 11,000, those that appeared infrequently or were considered to contribute minimally to the prediction of the outcome (i.e., certification of Care Level 2 or higher) were consolidated to enhance the efficiency of the machine learning process.

Specifically, 5,697 ICD-10-based diagnostic variables were subjected to exploratory machine learning using the Pointwise Linear Model (B3; Hitachi, Ltd., Tokyo, Japan) to identify those with significant regression coefficients (weights). The B3 model has been reported to perform well in detecting distinctive patterns even among low-frequency explanatory variables (16, 17). Diagnostic variables not selected through this process were categorized according to the Diagnosis Procedure Combination (DPC) system, a case-mix classification framework used in Japan’s hospital reimbursement system (18). As a result, 814 diagnosis-related explanatory variables were selected for inclusion in the main analysis.

In a similar manner, 6,069 procedure-related variables were consolidated into 252 representative variables. In contrast, all 690 medication-related and 621 long-term care service-related variables recorded in claims data were retained without consolidation. Additionally, health checkup data included 52 variables related to health behaviors, 21 variables on medical and medication history, and 123 variables derived from biochemical tests, all of which were included as separate explanatory variables. Through this process, a total of 2,753 explanatory variables were determined and linked at the individual level to the dependent variable for use in the main supervised machine learning analysis.

For the primary machine learning analysis using the training dataset, we employed Extreme Gradient Boosting (XGBoost), renowned for its high predictive accuracy and robustness in handling structured data (19). The model’s predictive performance was assessed by comparing its outputs with actual long-term care certification records for all individuals in the test dataset. The primary evaluation metric was the area under the receiver operating characteristic curve (AUC), complemented by sensitivity and specificity as secondary metrics. Additionally, the precision, F1 score and the area under the precision–recall curve (AUPRC) were calculated. In short, each subject’s most recent 21-month dataset was input into the model. Predicted risk probabilities were generated, and individuals with a risk score ≥0.5 were classified as high risk, while those with scores <0.5 were classified as low risk. These classifications were then matched against actual long-term care certification outcomes to compute the AUC, sensitivity, and specificity, thereby evaluating the model’s classification accuracy.

### Statistical Analysis

To assess the contribution of explanatory variables to the model’s predictive performance, permutation importance (PI) was calculated using AUC as the evaluation metric (20). Variables related to diagnoses, procedures, medications, and caregiving services were categorized according to the Diagnosis Procedure Combination (DPC) system, the Japan Standard Commodity Classification (21), and the Long-Term Care Insurance reimbursement classifications, respectively. PI was then computed for each category.

Furthermore, the marginal effects (ME) of 2,753 variables were estimated using probit regression analysis (22), with results expressed in percentage points. A positive ME value indicates an increase in the predicted risk, whereas a negative value suggests a decrease. The statistical significance of these marginal effects was evaluated using a stepwise selection method. All analyses were conducted using Python version 3.8.10 (https://www.python.org), XGBoost version 1.6.2 (https://github.com/dmlc/xgboost), and Stata version 17 (StataCorp LLC, College Station, TX, USA).

### Ethics

The Kobe City Health Bureau provided record-linked anonymized data. This study was conducted on an opt-out basis after approval by the Medical Ethics Committee of Kobe University (Approval No. B200234; September 19, 2020), the Kobe City Health Project Research Ethics Committee (March 22, 2021), and the Ethics Review Committee of the Hitachi Group (December 28, 2020). According to the ethical guidelines of the Hitachi Group, its members are permitted to participate in data analysis only when a given feature is present in more than ten individuals to minimize the risk of personal identification. Access to the dataset is restricted to the Kobe University laboratory to ensure data security and confidentiality.

## RESULTS

### Baseline characteristics of the analyzed subjects

Table I presents the baseline characteristics of the training and test datasets. Differences between the two datasets, particularly in gender, age, and care needs, reflect the sampling methods employed. Commonly observed conditions in both datasets included gastrointestinal, cardiovascular, musculoskeletal, and endocrine disorders, along with their corresponding treatments (Appendix 1).

### Machine learning model

The predictive performance of the supervised machine learning model for certification of Care Level 2 or higher was evaluated using a test dataset comprising 71,562 individuals, with a certification prevalence of 5.6%. The model achieved an AUC of 0.878, with a sensitivity of 0.784 and a specificity of 0.820. The precision, F1 score and AUPRC were 0.205, 0.325 and 0.359, respectively.

**Figure 1:**
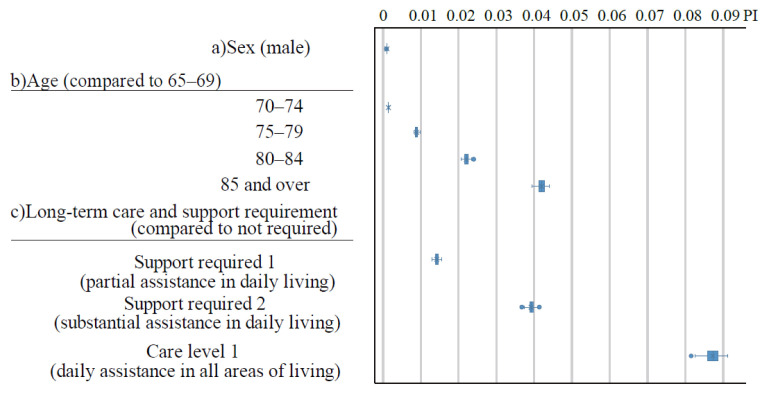
The feature importance of input variables related to sex (a), age (b), and long-term care and support requirements (c) in predicting Care level 2 and higher certification among the elderly. Each point and bar represent the mean value and the confidence interval (CI) of permutation importance (PI) in percentage points.

**Figure 2:**
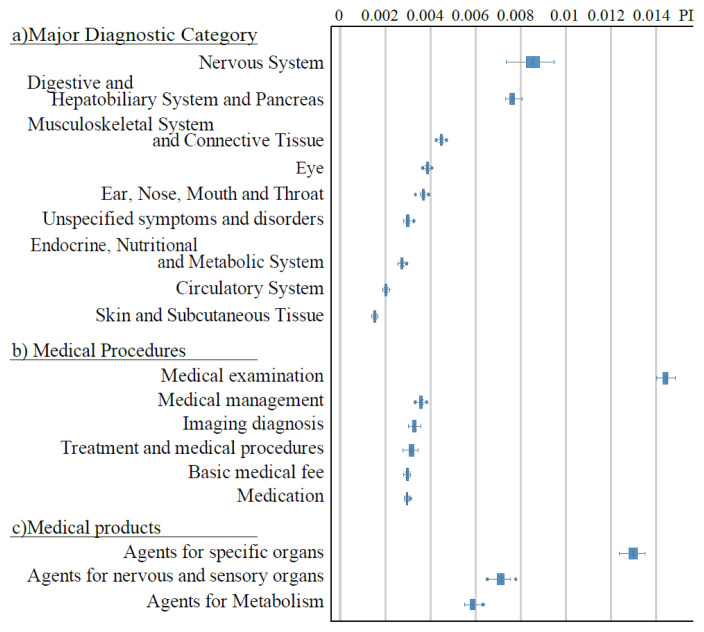
The feature importance of input variables related to major diagnostic categories (a), medical procedure (b), and medical products (c) in predicting Care level 2 and higher certification among the elderly. Each point and bar represent the mean value and the confidence interval of permutation importance (PI) in percentage points.

Figure 1 illustrates the permutation importance (PI) of explanatory variables related to gender, age, and care-needing status. These variables generally exhibited substantially higher PI values compared to other categories (Appendices 2–4), although gender demonstrated only a negligible contribution. Among major diagnostic, procedural and medical product categories, variables associated with neurological, gastrointestinal and musculoskeletal disorders, as well as medical examinations, showed relatively high PI values (Figure 2).

**Figure 3:**
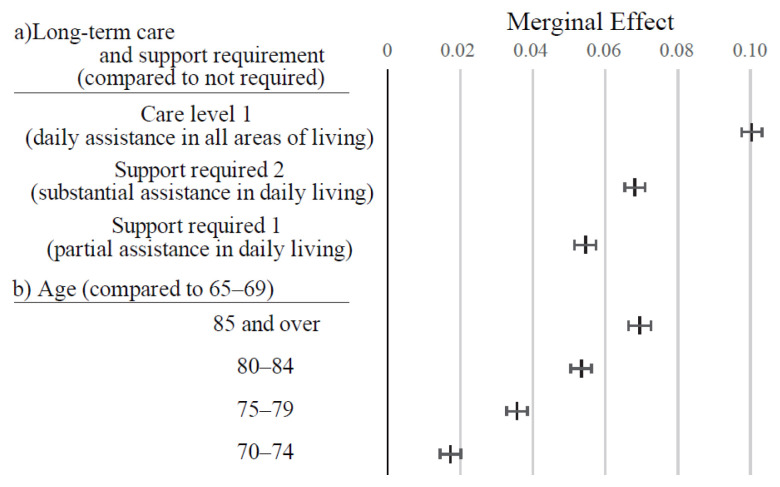
Marginal effects (ME) of input variables related to long-term care and support requirement and age (b) in predicting Care level 2 and higher certification among the elderly. Each point and bar represent the mean value and the confidence interval of ME in percentage points. All data were statistically significant by stepwise regression analysis.

**Figure 4:**
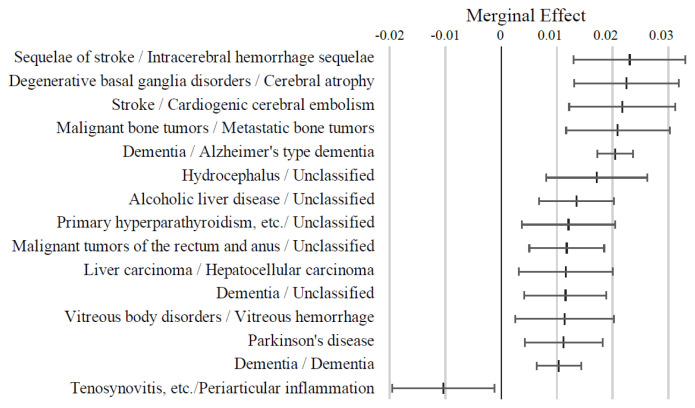
Marginal effects (ME) of input variables related to diagnostic code in predicting Care level 2 and higher certification among the elderly. Each point and bar represent the mean value and the confidence interval of ME in percentage points. All data were statistically significant by stepwise regression analysis.

**Figure 5:**
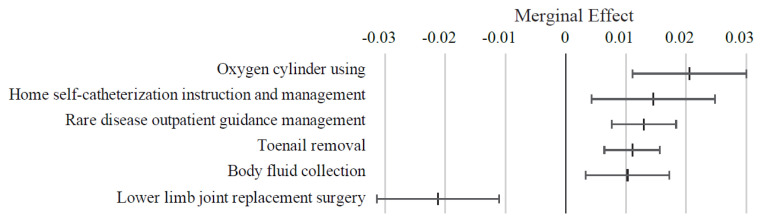
Marginal effects (ME) of input variables related to medical procedures in predicting Care level 2 and higher certification among the elderly. Each point and bar represent the mean value and the confidence interval of ME in percentage points. All data were statistically significant by stepwise regression analysis.

**Figure 6:**
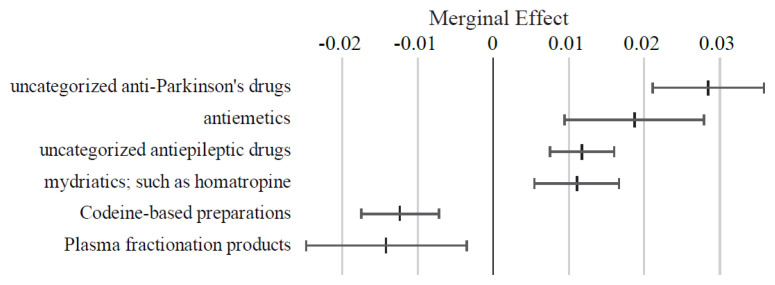
Marginal effects (ME) of input variables related to medical products in predicting Care level 2 and higher certification among the elderly. Each point and bar represent the mean value and the confidence interval of ME in percentage points. All data were statistically significant by stepwise regression analysis.

**Figure 7:**
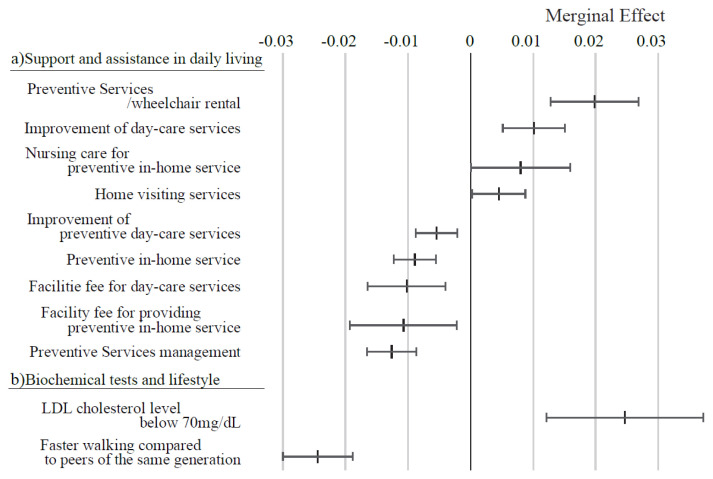
Marginal effects (ME) of input variables related to support and assistance in daily living (a) and biochemical tests and lifestyle (b) in predicting Care level 2 and higher certification among the elderly. Each point and bar represent the mean value and the confidence interval of ME in percentage points. All data were statistically significant by stepwise regression analysis.

Figures 3–7 and appendices 5–7 display explanatory variables with the statistically significant marginal effects (ME) derived from the probit regression analysis. Variables associated with care-needing status and advanced age exhibited the distinct high ME values (Figure 3). In particular, Care Level 1, Support Level 2, and age ≥85 showed ME values ranging from 6 to 10 percentage points.

As shown in Figure 4, variables associated with neurological, cardiovascular, gastrointestinal, musculoskeletal, and ocular conditions generally exhibited positive ME values. Notably, stroke, cerebral atrophy, metastatic bone tumors, and Alzheimer’s-type dementia were associated with ME values exceeding 2 percentage points, followed by hydrocephalus and alcoholic liver disease. In contrast, periarticular inflammation demonstrated a distinctive negative ME value. Although constipation, osteoporosis, and lower back pain had relatively small ME values of less than 1 percent point, each condition was observed in over 50,000 individuals out of the total study population of 284,660 subjects. Data on other diagnostic code variables are summarized in Appendices 5-1 and 5-2.

Figure 5 presents the ME values of variables related to medical procedures. Oxygen cylinder use, home self-catheterization instruction, outpatient management for rare diseases, toenail removal and body fluid collection were associated with positive ME values exceeding 1 percentage point. In contrast, procedures such as hip replacement surgery and percutaneous coronary intervention demonstrated negative ME values of approximately −2 percentage points. Most other variables in this category exhibited relatively small ME values (Appendices 6-1, 6-2 and 6-3).

As illustrated in Figure 6, medications such as uncategorized anti-Parkinsonian agents, antiemetics, uncategorized antiepileptic agents, and mydriatics (e.g., homatropine) were associated with positive ME values exceeding 1 percentage point. In contrast, medications with negative ME values below −1 percentage point included codeine-based preparations and plasma fractionation products. Other variables in this category exhibited relatively small ME values (Appendices 7-1 and 7-2).

Figure 7 displays the ME values of variables related to caregiving services. Wheelchair rental and day care service enhancements were associated with positive ME values exceeding 1 percentage point. In contrast, variables such as preventive service management, facility fees for in-home preventive services, and day care service fees demonstrated negative ME values below −1 percentage point. Other variables in this category exhibited relatively small ME values. Among variables associated with biochemical tests and lifestyle, a low-density lipoprotein (LDL) cholesterol level below 70 mg/dL was associated with a positive ME value exceeding 2 percentage points. In contrast, self-reported “faster walking compared to peers of the same generation” demonstrated a notable negative .ME value below −2 percentage points.

## DISCUSSION

This study is the first to demonstrate that data from Japan’s Community-based Health Insurance System can be used in machine learning-based prediction and risk factor identification of long-term care needs in the elderly. The developed model achieved a predictive accuracy of 0.878 (AUC), surpassing the internationally accepted benchmark for an effective classifier (AUC ≥ 0.8) (23). As both sensitivity and specificity were approximately 80%, the model demonstrates sufficient performance to warrant deployment in real-world settings. Although the precision (0.205) and F1 score (0.325) are modest due to the low prevalence of Care Level 2 certification (5.6%), such attenuation is inherent to screening tasks for rare outcomes. The AUPRC of 0.359, which adjusts for class imbalance, demonstrates the model’s capacity to rank individuals by risk with reasonable precision. Further optimization of decision thresholds and integration of cost-sensitive learning may improve its clinical applicability.

Analyses of PI and ME of the explanatory variables have revealed that Long-Term Care Level 2 certification is a multifactorial process influenced by a variety of risk and preventive factors. PI quantifies the contribution of each variable to the overall predictive performance of the model, whereas ME represents the directional impact of each variable on the predicted risk value. Variables related to old age and long-term care and support requirements had distinctive PI values and ME, indicating their considerable importance in risk prediction. Among major diagnosis categories, nervous system and gastrointestinal disorders and connective tissue impairments showed relatively higher PI values. ME analysis also revealed that the model assigned high ME values to several intractable conditions, including progressive and treatment-resistant central nervous system diseases such as dementia, stroke, gastrointestinal cancers, and fractures. These findings are broadly consistent with the results of analyses on medications and align with established knowledge regarding the widely recognized causes of long-term care needs (24–26). These consistencies support the reliability of our model in predicting the risk of the complex outcome of long-term care certification.

In the present PI analysis, the category of medical examination was noted to have a distinctive impact on predictive performance. Generally, medical examinations are conducted in relation to the diagnosis and treatment of serious disease conditions. Therefore, it is plausible that this variable functions as a proxy indicator for the severity of refractory chronic diseases, although further analysis is warranted. On the other hand, no difference in PI was observed between genders in this study. It is well established that elderly women are at higher risk of physical function decline than their male counterparts (27). One possible explanation for this discrepancy is that under-sampling during training dataset preparation may have resulted in balanced covariates, such as age distribution and disease composition, between the sexes.

The ME analysis also provided novel insights into previously unrecognized risk and protective factors for long-term care needs. Particularly, alcoholic hepatitis had a relatively high ME value, comparable to those of dementia and malignant tumors. To date, no reports have highlighted a relationship between long-term nursing care and alcoholic hepatitis. In contrast, diseases with established treatment options, such as periarticular inflammation and headaches, were found to exhibit significantly negative ME values. Distinctive negative ME value was also assigned to medications for chronic respiratory diseases. These findings suggest that appropriate treatment of conditions for which effective therapies are available may reduce the risk of Long-Term Care Level 2 certification. The reason why plasma fractionation products exhibited significant negative ME values remains unclear. Although further investigation is necessary, this finding may be associated with the use of these medications in the treatment of malignant cancers, advanced alcoholic hepatitis, and liver fibrosis.

Data on medical procedures indicated that variables related to the treatment of severe conditions, such as oxygen cylinder use and home self-catheterization instruction, were assigned large positive ME values. In contrast, procedures associated with interventions that promote independent living, such as lower limb joint replacement surgery, generally exhibited negative ME values. Furthermore, analysis of support and assistance services revealed that those aimed at addressing advanced physical function decline were associated with positive ME values, whereas early preventive interventions tended to show negative values. Taken together, these findings suggest that interventions focusing on early recovery and the maintenance of independent living may be effective in preventing progression to Long-Term Care Level 2 certification. To support such preventive efforts, this model may serve as a practical tool for municipal health authorities and primary care providers to identify high-risk individuals and initiate timely preventive care interventions. However, this study was conducted as an observational analysis aimed at risk prediction rather than the estimation of causal effects. The use of a large, heterogeneous, and comprehensive dataset inevitably imposed constraints on covariate selection and adjustment, which may have introduced residual confounding. Consequently, the marginal effects estimated by our model should be interpreted as indicative of associations, not direct causal effects. To advance the evidence base and inform effective public health interventions, further research employing robust causal inference methodologies is essential to determine whether the associations between various interventions and Long-Term Care Level 2 certification observed in this study reflect genuine causal relationships.

For the factors related to biochemical tests and lifestyle, low LDL cholesterol (LDL-C) levels were associated with distinctively high ME values. This finding is consistent with previous meta-analyses indicating that elderly individuals with higher LDL-C levels tend to have equal or greater longevity compared to those with lower LDL-C levels (28, 29). In contrast, gait speed exhibited a large negative ME value. This result aligns with prior evidence from a 20-year prospective cohort study demonstrating a significant association between slower gait speed and the onset of disability (30). Our finding further supports the utility of gait speed as a predictive indicator for assessing the risk of functional decline and loss of independence.

In summary, we developed a machine learning model to assess the individual-level risk of long-term nursing care needs in the elderly. This model enables objective, data-driven screening to identify individuals at elevated risk, thereby supporting healthcare professionals in enhancing the efficiency of both population-level interventions and personalized preventive strategies aimed at extending healthy life expectancy.

While machine learning is well-suited for uncovering latent patterns within large datasets, its capacity to establish causal relationships remains limited. Therefore, further studies, including subgroup analyses, are required to clarify the precise associations between long-term care needs and the risk factors identified in this study. Additionally, achieving “explainability”, the ability to present risk factors alongside predicted risk scores is essential for applying our model to sensitive contexts such as clinical decision-making. Collectively, however, the present study underscores the potential of machine learning to guide policy-level planning and support personalized care, promoting efficient long-term care management in an aging society.

## Figures and Tables

**Appendix 2:**
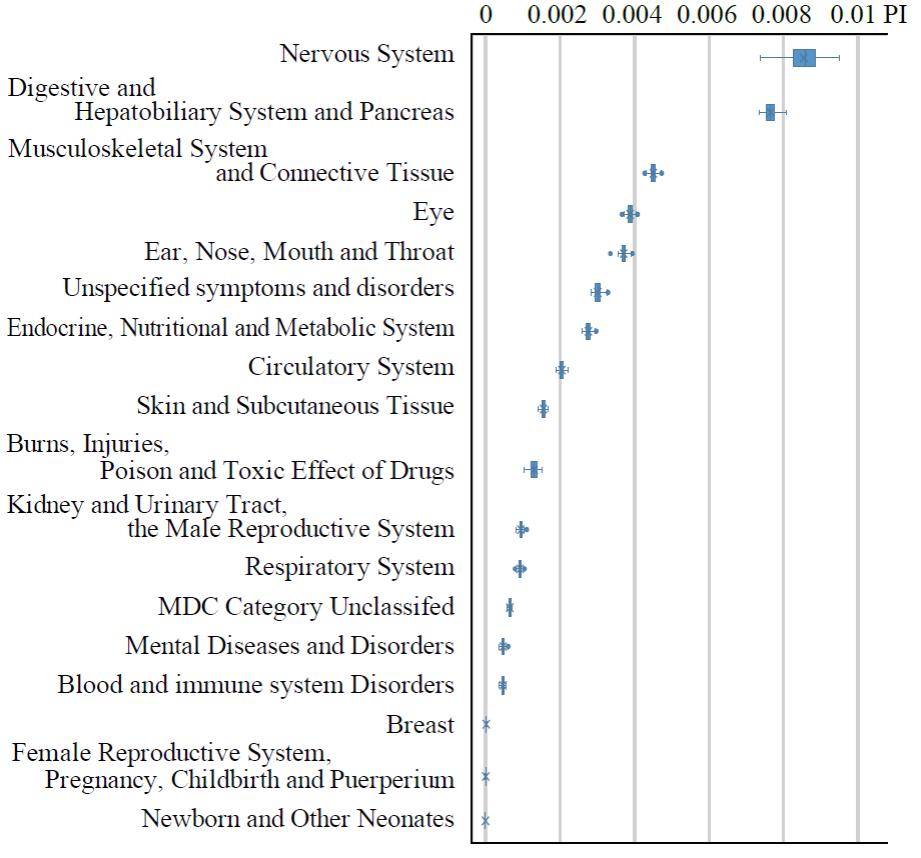
The feature importance of input variables related to major diagnostic categories in predicting Care level 2 and higher certification among the elderly. Each point and bar represent the mean value and the confidence interval of permutation importance (PI) in percentage points. Part of the data is shown in Figure 2 in the main text.

**Appendix 3:**
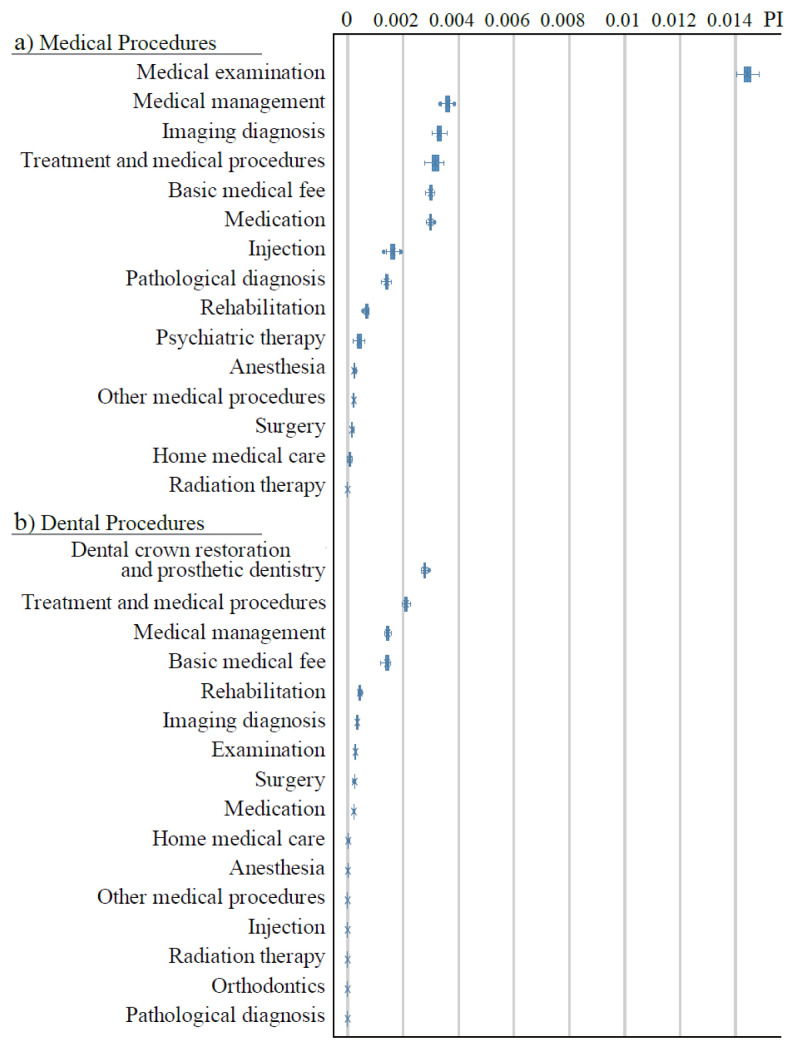
The feature importance of input variables related to medical (a) and dental (b) procedures in predicting Care level 2 and higher certification among the elderly. Each point and bar represent the mean value and the confidence interval of permutation importance (PI) in percentage points. Part of the data is shown in Figure 2 in the main text.

**Appendix 4:**
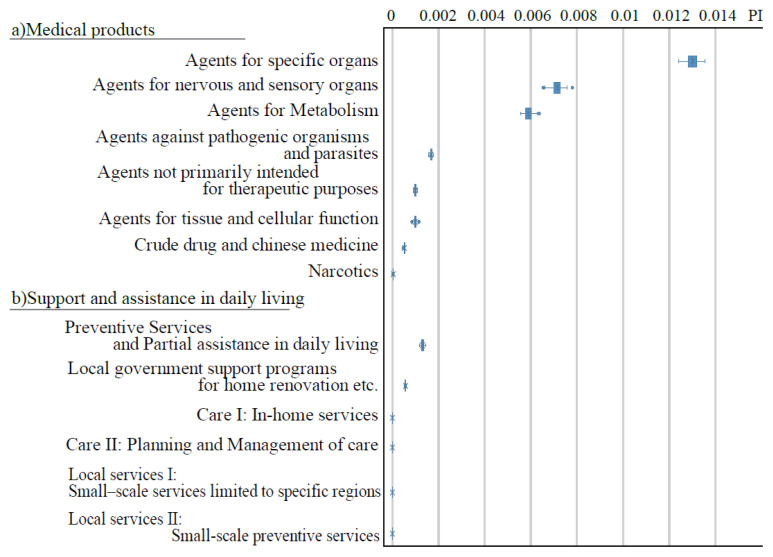
The feature importance of input variables related to medical products (a) and support and assistance in daily living (b) in predicting Care level 2 and higher certification among the elderly. Each point and bar represent the mean value and the confidence interval of permutation importance (PI) in percentage points. Part of the data is shown in Figure 2 in the main text.

**Appendix 5-1:**
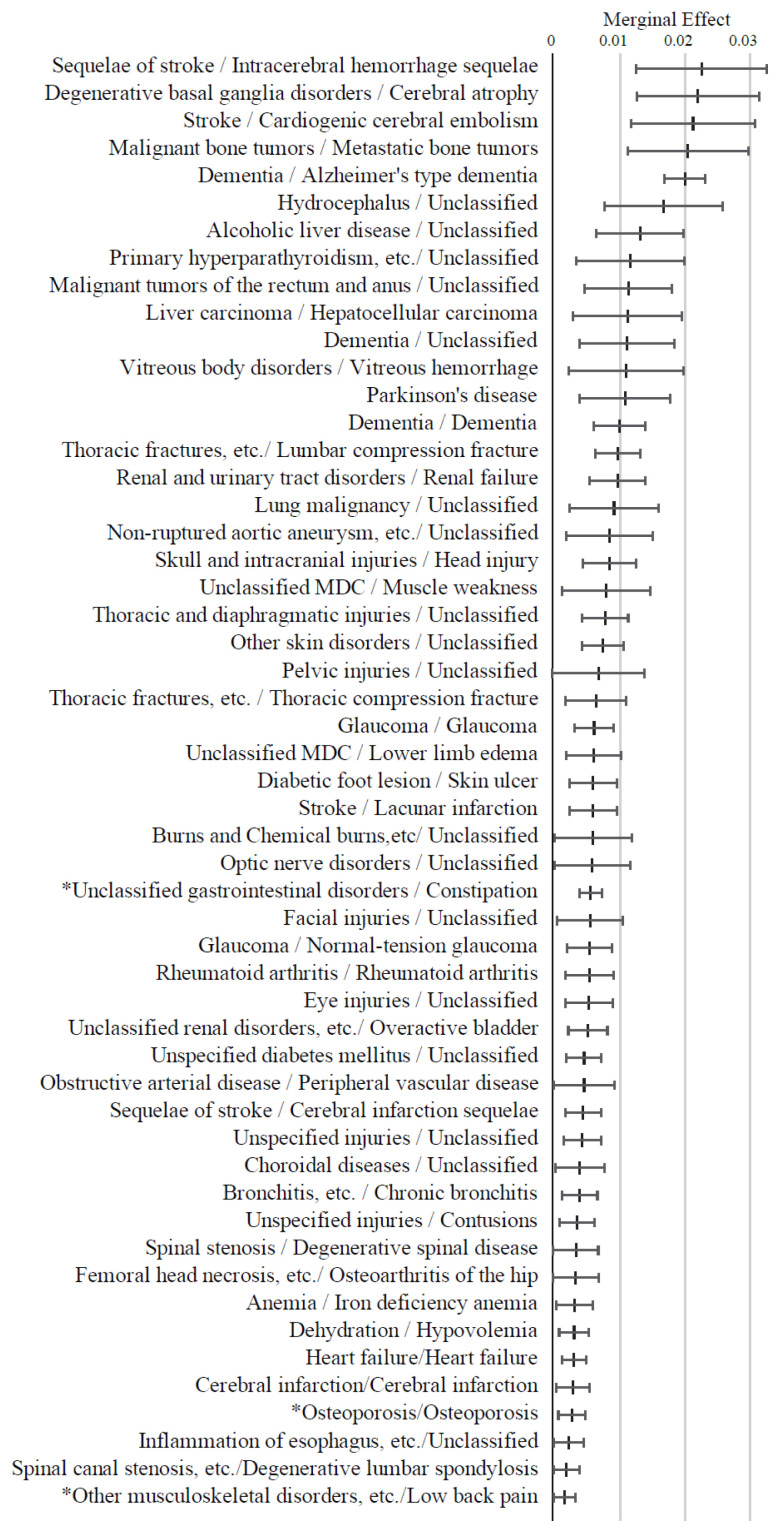
Variables with positive marginal effects (ME) values related to diagnostic code in predicting Care level 2 and higher certification among the elderly. Each point and bar represent the mean value and the confidence interval of ME in percentage points. All data were statistically significant by stepwise regression analysis. Part of the data is shown in Figure 4 in the main text.

**Appendix 5-2:**
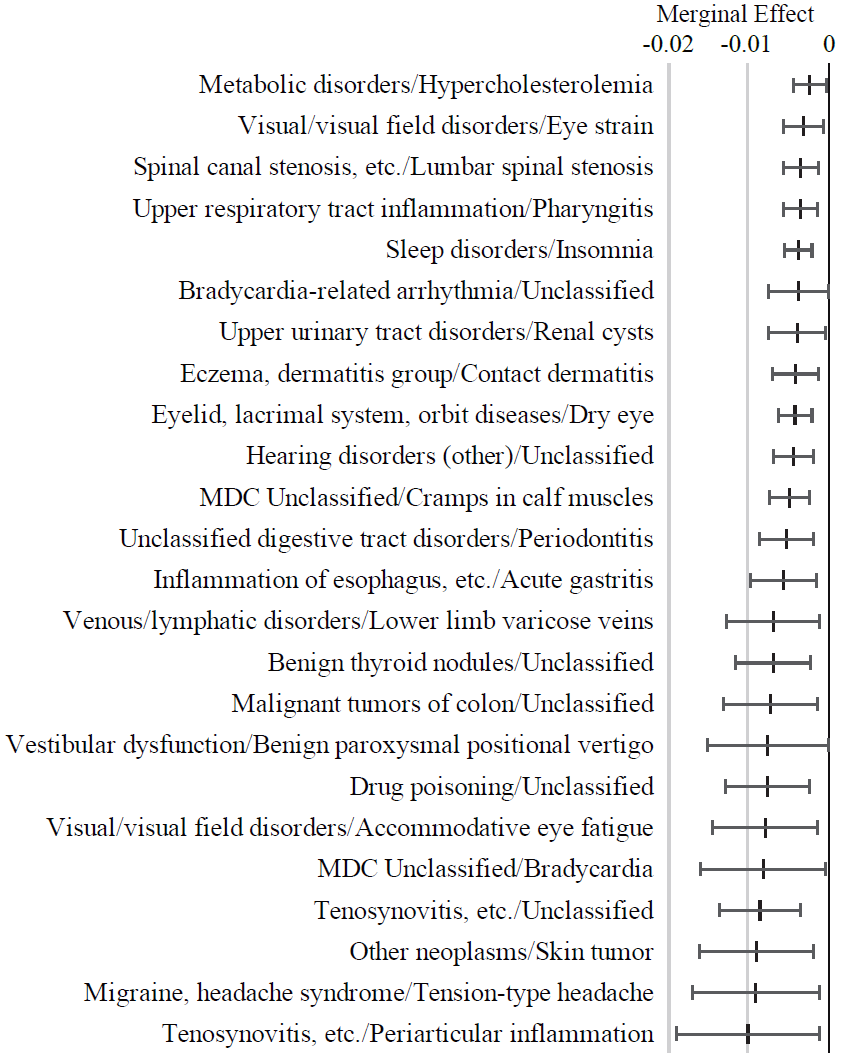
Variables with negative marginal effects (ME) values related to diagnostic code in predicting Care level 2 and higher certification among the elderly. Each point and bar represent the mean value and the confidence interval of ME in percentage points. All data were statistically significant by stepwise regression analysis. Part of the data is shown in Figure 4 in the main text.

**Appendix 6-1:**
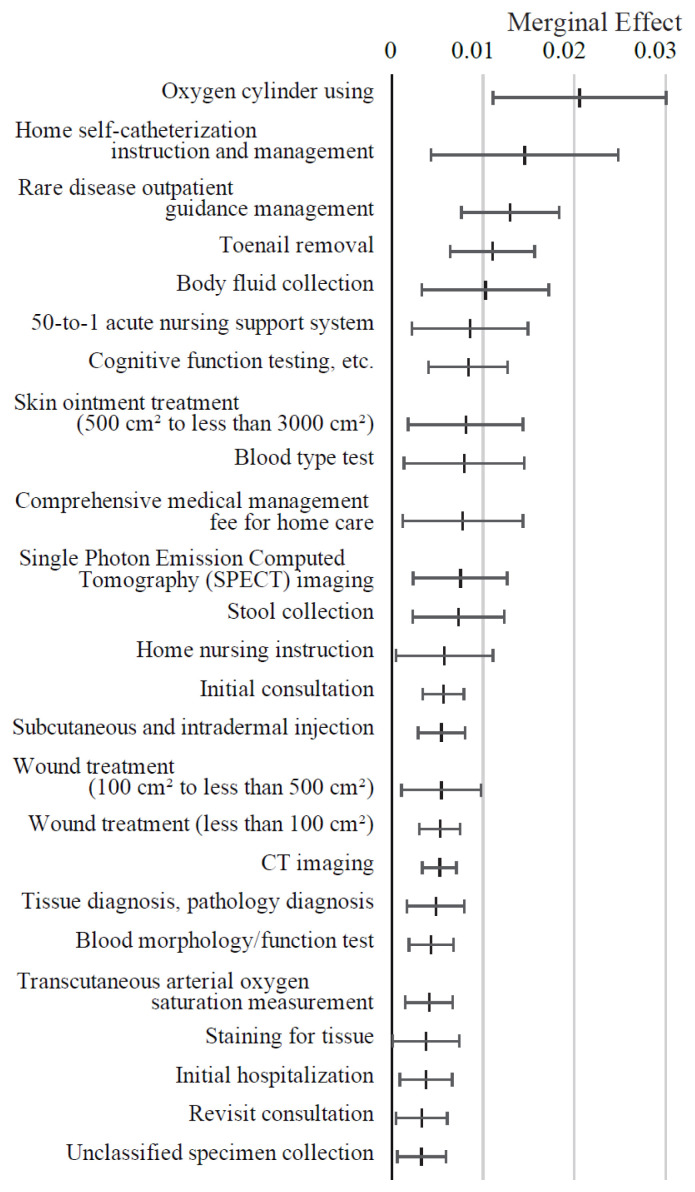
Variables with positive marginal effects (ME) values related to medical procedures in predicting Care level 2 and higher certification among the elderly. Each point and bar represent the mean value and the confidence interval of ME in percentage points. All data were statistically significant by stepwise regression analysis. Part of the data is shown in Figure 5 in the main text.

**Appendix 6-2:**
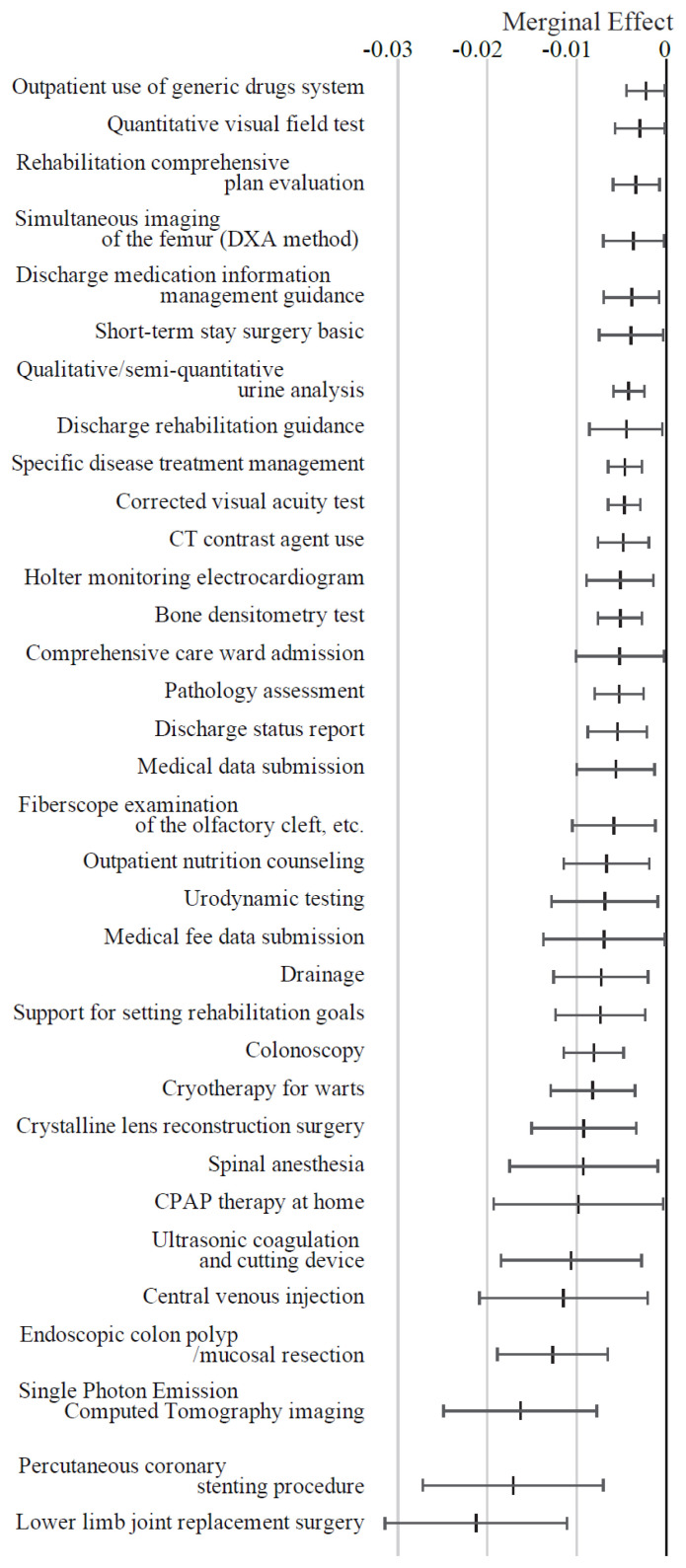
Variables with negative marginal effects (ME) values related to medical procedures in predicting Care level 2 and higher certification among the elderly. Each point and bar represent the mean value and the confidence interval of ME in percentage points. All data were statistically significant by stepwise regression analysis. Part of the data is shown in Figure 5 in the main text.

**Appendix 6-3:**
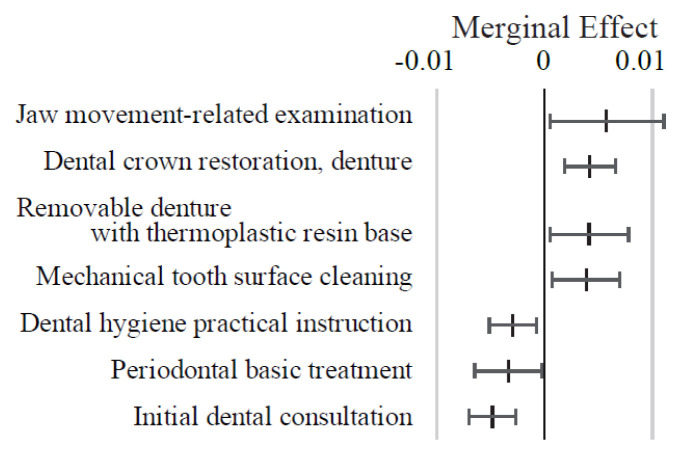
Marginal effects (ME) related to dental procedures in predicting Care level 2 and higher certification among the elderly. Each point and bar represent the mean value and the confidence interval of ME in percentage points. All data were statistically significant by stepwise regression analysis. All data were statistically significant by stepwise regression analysis.

**Appendix 7-1:**
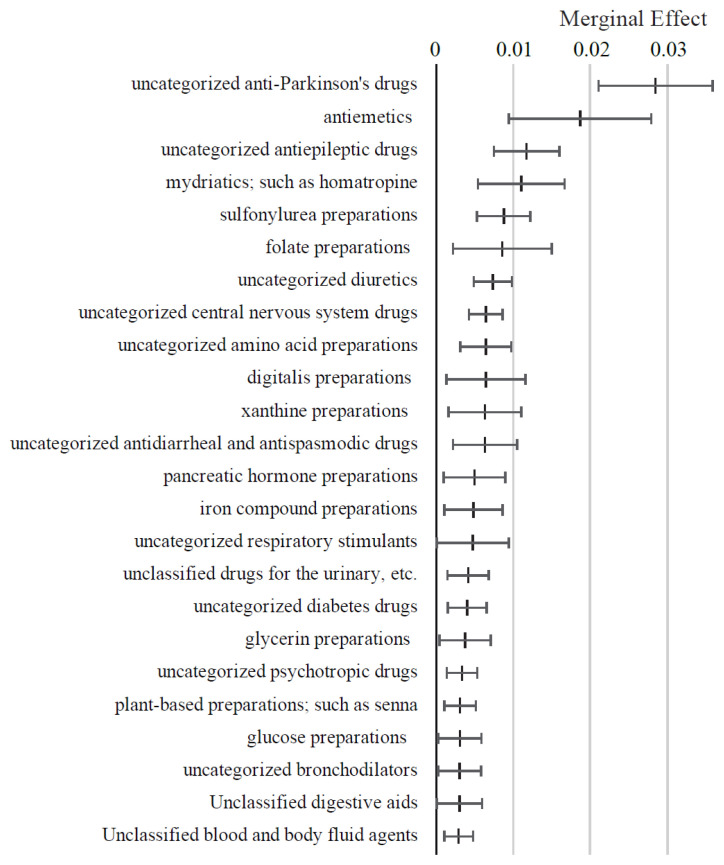
Variables with positive marginal effects (ME) related to medical products in predicting Care level 2 and higher certification among the elderly. Each point and bar represent the mean value and the confidence interval of ME in percentage points. All data were statistically significant by stepwise regression analysis. Part of the data is shown in Figure 6 in the main text.

**Appendix 7-2:**
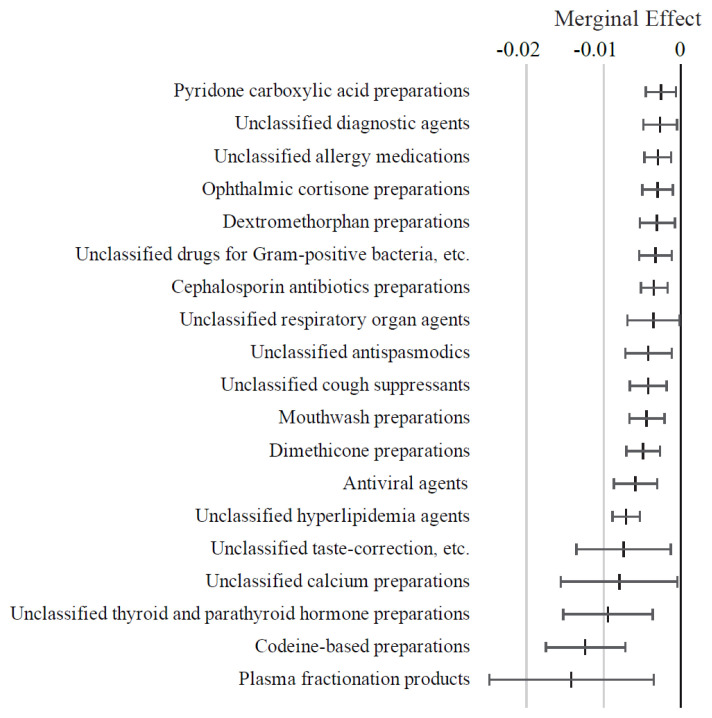
Variables with negative marginal effects (ME) related to medical products in predicting Care level 2 and higher certification among the elderly. Each point and bar represent the mean value and the confidence interval of ME in percentage points. All data were statistically significant by stepwise regression analysis. Part of the data is shown in Figure 6 in the main text.

**Table I:** Base line characterisiteics of the training and testing dataset

		Training dataset N = 23,878		Testing dataset N = 71,562	
		Case	Rate	Case	Rate
Sex	Male	9,168	38.4	29,292	40.9
Age	65–69	4,223	17.7	20,025	28.0
	70–74	4,386	18.4	17,876	25.0
	75–79	5,090	21.3	16,244	22.7
	80–84	5,321	22.3	11,169	15.6
	85 and over	4,857	20.3	6,248	8.7
Long-term care and support requirement certification					
	Not Certified	15,316	64.1	62,451	87.3
	Support required 1	1,645	6.9	2,646	3.7
	Support required 2	3,188	13.4	3,961	5.5
	Care level 1	3,729	15.6	2,504	3.5

**Appendix 1:** Detailed baseline characteristics of the training and testing data. Part of the data is shown in Table I in the main text.

	Training dataset N = 23,878		Testing dataset N = 71,562	
	Case	Rate	Case	Rate
Sex and Age				
Sex (male)	9,168	38.4	29,292	40.9
Age 65–69	4,224	17.7	20,025	28.0
70–74	4,386	18.4	17,876	25.0
75–79	5,090	21.3	16,244	22.7
80–84	5,321	22.3	11,169	15.6
85 and over	4,857	20.3	6,248	8.7
Long-term care and support requirement certification				
Not certified	15,316	64.1	62,451	87.3
Support required 1 (partial assistance in daily living)	1,645	6.9	2,646	3.7
Support required 2 (substantial assistance in daily living)	3,188	13.4	3,961	5.5
Care level 1 (daily assistance in all areas of living)	3,729	15.6	2,504	3.5
Major Diagnostic Categories				
Digestive and Hepatobiliary System and Pancreas	21,671	90.8	64,430	90.0
Circulatory System	18,797	78.7	51,486	71.9
Musculoskeletal System and Connective Tissue	17,883	74.9	49,256	68.8
Unspecified symptoms and disorders	17,703	74.1	48,973	68.4
Endocrine, Nutritional and Metabolic System	17,561	73.5	49,504	69.2
Nervous System	15,400	64.5	38,030	53.1
Ear, Nose, Mouth and Throat	14,720	61.6	42,945	60.0
Eye	13,511	56.6	40,053	56.0
Skin and Subcutaneous Tissue	12,395	51.9	33,590	46.9
MDC Category Unclassified	12,149	50.9	34,701	48.5
Respiratory System	10,780	45.1	29,402	41.1
Burns, Injuries, Poison and Toxic Effect of Drugs	9,979	41.8	24,039	33.6
Kidney And Urinary Tract, the Male Reproductive System	9,298	38.9	23,446	32.8
Mental Diseases and Disorders	6,392	26.8	14,353	20.1
Blood and immune system Disorders	5,171	21.7	11,326	15.8
Female Reproductive System, Pregnancy, Childbirth and Puerperium	941	3.9	2,774	3.9
Breast	710	3.0	2,215	3.1
Newborn And Other Neonates	356	1.5	937	1.3
Support and assistance in daily living				
Local services : Small-scale preventive services	36	0.2	32	0.04
Preventive Services and Partial assistance in daily living	4,480	18.8	5,826	8.1
Local government support programs for home renovation etc.	2,273	9.5	3,258	4.6
